# 

**Published:** 1868-04

**Authors:** 


### Books and Pamphlets Received.

*A Manual of the Dissection of the Human Body. By Luther Holden, F. F. C. S., Assistant Surgeon of and Lecturer on Anatomy at St. Bartholomew's Hospital, London, with notes and additions by Erskine Mason, M. D., Demonstrator of Anatomy at the College of Physicians and Surgeons, etc., illustrated with numerous wood engravings. New York: Robert M. De Witt, Publisher, No.* 13 *Frankfort street.*

*Contributions to the Causation and Prevention of Disease and to Camp Diseases, together with a report of the Diseases, etc., among the Prisoners at Andersonville, Ga. Edited by Austin Flint, M. D., New York. Published by the U. S. Sanitary Commission, by Hurd & Houghton,* 459 *Broome street,* 1867. *For sale by Martin Taylor.*

*The Endoscope and its Application to the Diagnosis and Treatment of Affections of the Genito-Urinary Passages. Lessons given at Necker Hospital, by A. J. Desormeaux, Surgeon of the hospital, etc. Translated by R. P. Hunt, M. D.*

*Address delivered before the Philadelphia County Medical Society, by William Maybury, M. D., at the close of his official term as President.*

*Report of the Massachusetts General Hospital for the year* 1867.

*Report of the Buffalo General Hospital for the year* 1867.

*Annual Report of the Board of Health of the City of Toledo for the year* 1867.

